# Evaluation of Type 1 Error Rates in Duplex Sequencing for Mutagenicity Testing Using Vehicle Control Data and Simulation Analyses

**DOI:** 10.1002/em.70067

**Published:** 2026-06-16

**Authors:** Andrew Williams, Shaofei Zhang, Dingzhou Li, Wen Sun, Maik J. Schuler, Francesco Marchetti, Carole L. Yauk

**Affiliations:** ^1^ Environmental Health Science and Research Bureau, Health Canada Ottawa Ontario Canada; ^2^ Pfizer Worldwide Research, Development, and Medical Groton Connecticut USA; ^3^ Nonclinical Safety, Bristol Myers Squibb New Brunswick New Jersey USA; ^4^ Department of Biology University of Ottawa Ottawa Ontario Canada

**Keywords:** duplex sequencing, mutagenicity testing, type 1 error

## Abstract

Accurate mutation detection and quantification are crucial for understanding mutagenesis and its potential health implications. Traditional in vivo mutagenicity assays, such as the transgenic rodent gene mutation assay, are limited by their focus on single reporter genes and inability to efficiently generate mutation spectra. Error‐corrected sequencing (ECS) technologies like Duplex Sequencing (DS) offer significant advantages, including extremely low error rates and the ability to measure mutation frequencies (MFs) across various tissues and model organisms. Before ECS approaches can be adopted for regulatory purposes, their performance characteristics, particularly the type 1 error rate, must be rigorously established. We evaluated the type 1 error rate of DS through empirical analysis of vehicle control data and complementary simulation studies. Using 138 control mouse liver samples from 28 studies analyzed with the TwinStrand Mouse Mutagenesis Panel, we performed variance component analysis and found that experiment‐level variability exceeds within‐experiment sample variability. To evaluate the impact of between‐study heterogeneity, we simulated overdispersed binomial data informed by the observed variance components. Removing the most variable studies reduced overdispersion and improved control of the type 1 error rate. Our findings demonstrate that DS maintains appropriate type 1 error rates (~0.05) when study heterogeneity is limited and at least four samples per group are used. Under greater overdispersion, sample sizes of five or six per group may be needed to achieve comparable control of the type 1 error rate. These results underscore the importance of combining empirical and simulation‐based approaches to evaluate and optimize the statistical performance of emerging genomic technologies.

## 1 Introduction

Accurate quantification of mutations is fundamental to understanding mutagenesis and its potential implications for human health. Conventional in vivo mutagenicity assays, such as the transgenic rodent (TGR) gene mutation assay (OECD TG 488 2025a), have been used for this purpose for decades. The TGR assay, the established standard for in vivo mutation detection, relies on bacterial or viral transgenes that are integrated into the rodent genome. After exposures, these transgenes are recovered and packaged into viral phages. Mutants are then identified by plaque formation in bacterial lawns grown under selective conditions (Lambert et al. 2005).

While effective, these assays are limited by their focus on single reporter genes that do not fully represent the complexities of the mammalian genome, and by the fact that generating mutation spectra, though possible, is laborious and typically only a very minimal number of plaques are sequenced to evaluate changes in mutation spectrum. As a result, this aspect of the assay is rarely undertaken. Mutation spectra yield critical insights into mutagenic mechanisms (Beal et al. 2020) and can provide support for a mutagenic effect when the increase in mutations is small. Additionally, these stand‐alone assays are strain‐specific, limiting their applicability across different model organisms and integration into standard toxicity tests. These limitations highlight the need for more comprehensive and scalable approaches that better reflect the biological complexity of in vivo mutagenicity.

Error‐corrected sequencing (ECS) technologies, such as Duplex Sequencing (DS) (Salk and Kennedy 2020; Valentine et al. 2020), have emerged as powerful alternatives that address many limitations of traditional reporter‐based assays (Yauk et al. 2026; Marchetti et al. 2023; Menon and Brash 2023). DS is capable of quantifying mutation frequencies (MFs) across various tissue types and model organisms with high accuracy, as it uses specialized strategies to identify and remove artifactual (false) mutations from the analysis, thereby reliably detecting real mutations (Dodge et al. 2023; LeBlanc et al. 2022). Recent work by a multi‐laboratory collaboration has demonstrated that DS can be successfully implemented across laboratories with varying levels of experience and is capable of producing highly reproducible and accurate measures of mutagenicity (Zhang et al. 2025). While these results underscore the technical robustness of DS, further evaluation of its performance characteristics, including its type 1 error rate, is essential before it can be widely adopted.

The type 1 error rate describes how often a test incorrectly identifies a chemical as mutagenic when, in fact, it is not (i.e., the probability of a false positive result). This metric is important for evaluating assay validity, as an elevated type 1 error rate indicates that the test may generate an excess of false‐positive findings, leading to inappropriate rejection of non‐mutagenic compounds. Conversely, if statistical significance thresholds are too stringent, making true effects difficult to detect, the assay might miss genuine mutagenic responses, resulting in false negatives. This reduces the assay sensitivity and limits its ability to reliably identify hazardous substances. Balancing these error rates ensures that the assay is both robust and fit for regulatory decision‐making (OECD TG 488 2025a; Dertinger et al. 2023).

In this study, we directly measured how often DS might incorrectly identify a chemical as mutagenic using both real‐world data and computer simulations. We started by looking at untreated (vehicle control) mice, which reflect the background MF. This was then used to build computer models to simulate many experimental conditions (e.g., modifying sample size). By comparing results from both empirical datasets and simulations, we evaluate how study design choices and inter‐group variability can influence the accuracy and reliability of the test. This approach supports optimization of DS‐based mutagenicity testing by minimizing the risk of false‐positive findings, while maintaining appropriate sensitivity to detect true effects. Together, these considerations help ensure that the assay is both robust and fit for regulatory decision‐making.

## 2 Methods

### 2.1 Animals and Tissue Collection

A total of 138 male Big Blue C57BL/6 transgenic mice, sourced from Taconic Laboratories for Gentronix Ltd. (Alderly Park, UK), were utilized in this study, which was performed at Pfizer, Groton, CT, USA. They represent the vehicle control mice of 28 studies conducted between June 17, 2022, and April 24, 2025, at approximately monthly to quarterly intervals. All procedures performed on animals were in accordance with regulations and established guidelines and were reviewed and approved by the Pfizer Institutional Animal Care and Use Committee.

At the beginning of each study, mice were 8–12 weeks old and housed under controlled conditions: temperature maintained at 68°–79°F, relative humidity at 30%–70%, and a 12‐h light/dark cycle. Animals were provided ad libitum access to municipal drinking water purified by reverse osmosis and fed Certified Irradiated Rodent Diet 2916C (Envigo Teklad Global Diet).

At least five mice per vehicle control group were dosed by oral gavage once daily for 28 consecutive days. Vehicle controls and dose volumes are shown in Table S1. Individual dose volumes were calculated based on the animal's most recently recorded body weight. All animals were euthanized by isoflurane anesthesia followed by exsanguination on Day 31 and necropsied. Livers were collected, blotted to remove excess blood, flash frozen in liquid nitrogen and stored at −80°C until further processing for DNA extraction. The 28‐day exposure and 3‐day sampling time point adhere to the recommendations for mutation analysis in OECD Test Guideline 488 (OECD TG 488) and have been successfully used for DS analyses in previous studies (Yauk et al. 2026).

### 2.2 DNA Extraction and Library Preparation for Duplex Sequencing

Genomic DNA was extracted from livers of at least five mice per group using DNeasy Blood & Tissue Kits (QIAGEN, catalog# 69504 or 69506). Approximately 500 ng of extracted DNA from each animal was fragmented to a median size of about 200 to 300 base pairs with TwinStrand DuplexSeqTM Kit‐Enzymatic Fragmentation Module. Libraries were then constructed from enzymatically digested DNA using the DuplexSeq Mouse Mutagenesis kit (TwinStrand Biosciences Inc., Seattle, WA). Library preparation involved end‐repair and A‐tailing, followed by ligation of DuplexSeq adapters containing unique molecular identifiers (UMIs). DNA target regions were enriched via hybrid capture using the probes supplied by the Mouse Mutagenesis kit, then amplified via PCR using reagents provided in the kit. The final libraries were quantified using a Qubit fluorometer and the Agilent TapeStation system (to confirm size of the fragments). Pooled samples were sequenced on Illumina NovaSeq 2x150bp systems (NovaSeq 6000 or NovaSeq X) at Azenta Life Sciences (South Plainfield, NJ).

The FASTQ files generated from sequencing were analyzed with the TwinStrand DuplexSeq Mutagenesis App on the DNAnexus cloud computing platform. Consensus calling and postprocessing, variant calling, and interpretation were performed as previously described (Valentine et al. 2020). The MF was calculated as the ratio between unique mutation count and the total duplex bases (excluding no‐calls) observed in the targeted regions (MFmin) as this metric is a conservative estimate of mutation burden and is recommended by an expert working group within the International Workshops on Genotoxicity testing (Yauk et al. 2026). The processed data are provided as Table S2 and the raw data are available upon request.

A threshold of 500 million consensus duplex bases per sample has been recommended as sufficient for reliable mutation frequency estimation in ECS studies, based on published power analyses and recent consensus recommendations (Dodge et al. 2023; Esina et al. 2024; Yauk et al. 2026). However, in line with IWGT guidance, samples with slightly lower denominators were included if quality metrics confirmed data reliability, as excluding them could reduce statistical power or bias results [Dertinger et al. 2023]. All included samples met these benchmarks for duplex consensus accuracy.

### 2.3 Selection and Characterization of Vehicle Control Data

To empirically evaluate the type I error rate of DS, we sought to identify a relatively homogeneous subset of vehicle control studies in which MF values were sufficiently similar across animals and studies to allow meaningful comparison. We used tools recommended by Dertinger et al. (2023) to characterize the variability structure of the control data, including: (a) control charts (Nelson 1984; Western Electric Company 1956) to visualize sample‐ and study‐level trends and identify outlying studies; (b) variance component analysis to quantify the relative contributions of sample‐ and experiment‐level variability. Control charts were generated in R using the “qcc package” (Scrucca 2004), and variance component analysis was conducted using the “lme4 package” (Bates et al. 2015).

Rather than excluding studies that showed greater variability or a wider spread in their MF data, we used these tools to partition the dataset into a relatively stable core group suitable for empirical evaluation and a more heterogeneous group to inform simulation scenarios with increased overdispersion. This strategy allowed us to both anchor our assessment of type 1 error under ideal conditions and explore how increasing variability impacts false discovery control.

### 2.4 Empirical Evaluation of the Type 1 Error

We empirically evaluated the type 1 error rate using vehicle control data, following an approach similar to that described by Lee et al. (2024). To simulate a situation where no real difference should exist, we took samples from the pool of vehicle control animals and randomly split them into two groups. One group was labeled “control” and the other labeled “treated.” Because both groups come from animals that were not exposed to any mutagen, we expect them to be the same. Our analysis then tests whether, by chance, we would see a statistical difference between these two otherwise identical groups. Whenever a difference is detected, it counts as a false positive result, meaning the test incorrectly suggests that a treatment effect exists when there actually is not one. Group sizes ranged from 3 to 20 to evaluate the impact of sample size on the type 1 error rate and to assess how the test statistic behaves as group sizes increase.

Group comparisons were conducted using a generalized linear model with a quasibinomial error structure to account for overdispersion. We chose this approach because generalized linear models (GLMs) are well‐suited for count‐based data (such as MF), handle the binomial nature of mutation data, and can flexibly account for extra variability beyond what is expected by simpler models like the *t*‐test or a standard binomial test. GLMs (and, where applicable, generalized linear mixed models (GLMMs)) also allow us to include both fixed effects (such as group differences) and random effects (such as variation across animals or experiments), which is important for accurately modeling the structure and extra variation often present in mutagenicity studies using ECS. This enables us to estimate type 1 error rates accurately, even when the data do not follow normal distributions. Statistical significance was assessed using the Wald statistic, as implemented in the doBy R package (Højsgaard and Halekoh 2023). For each group size, 10,000 simulation replicates were performed, and the type 1 error rate was estimated as the proportion of replicates yielding a *p*‐value below 0.05. To assess how much simulation results can fluctuate due to random variation (Monte Carlo error), each simulation scenario was independently repeated 10 times. Reporting the mean and confidence intervals across these runs helps show the reliability and stability of our error rate estimates; these values are presented in Figure 3.

### 2.5 Simulation Analysis

Overdispersion occurs when the observed variance exceeds what is expected under a standard binomial distribution; this is often due to heterogeneity among subjects, or animals, in our case. To account for this, we used a GLMM to simulate overdispersed binomial data, consistent with the methodologies described by Gelman and Hill (2007), Bolker (2008), and Johnson et al. (2015). The procedures for simulating overdispersion and estimating error rates closely follow those established previously in Esina et al. (2024). For clarity, all discussions regarding statistical power and optimal sample size are drawn from Esina et al. (2024), where comprehensive power analyses for DS mutagenicity studies were performed using similar GLMM‐based simulation approaches; the power results in the present manuscript are thus not new findings.

The simulation workflow for type 1 error analysis is as follows:

1. *Data Generation*: For each simulation, datasets were generated under an overdispersed binomial distribution using a random intercept model:
$$\;\text{logit}\left(p_{i}\right)=\log\left(\frac{p_{i}}{1-p_{i}}\right)=\mu +Z_{i}$$
  where $Z_{i}$ ~ $N\left(0,\sigma^{2}\right)$ represents inter‐sample variability, and the effect of interest (log fold change) was set to 0 (i.e., equivalent MF, or fold change of 1, between mock groups).
2. *Hypothesis Testing*: For each simulated dataset, group differences were evaluated using a Wald test within a quasibinomial GLM framework.
3. *Error Rate Estimation*: The type 1 error rate was estimated as the proportion of simulations yielding a statistically significant difference (*p* < 0.05) between vehicle control groups of mice that hypothetically should not be different from each other.

Simulation inputs included: (1) an estimate of MF, (2) the variance component, *σ* ^2^, capturing overdispersion among samples, and (3) the sample size. These parameters, as well as the data generation and statistical testing procedures, were based on those described by Esina et al. (2024), with the fold change parameter set to 1 (indicating no true difference).

## 3 Results and Discussion

**FIGURE 1:**
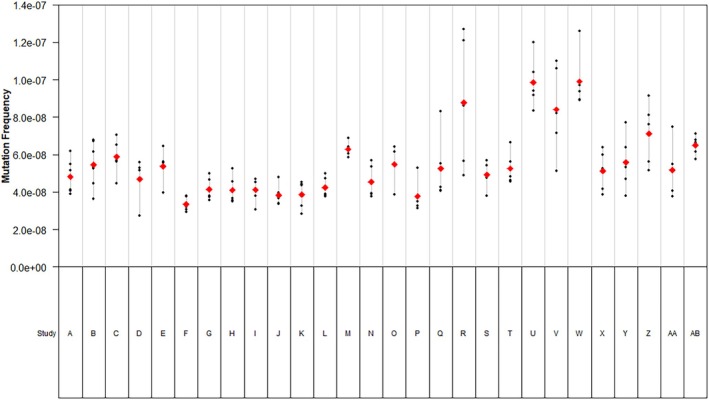
Variability chart of mutation frequencies in liver samples from inbred Big Blue mice across 28 Duplex Sequencing vehicle control studies. The variability chart is a dot plot with the black dots representing the observed mutation frequencies for each sample and the red diamonds representing the mean of the samples within each experiment. This figure was generated using the varPlot function in the VCA R library (Schuetzenmeister and Dufey 2024).

We evaluated vehicle control data comprising 28 studies on mouse liver mutagenesis using DS (138 control animals) as the basis to conduct simulation studies to define the type 1 error rate. This is a foundational analysis necessary to determine suitable study designs for DS analysis of mouse liver for regulatory mutagenicity assessment. All experiments had at least 5 animals in their respective vehicle control groups; experiments A and B had 6 and 7, respectively. Figure 1 graphically displays these data using a variability chart in chronological order. Variability charts are graphs that help to identify and understand where the differences in the data come from such as whether most of the variation is between individual animals or between different studies. The chart shows that the variation in the means of the experiments range from 3.4 × 10^−8^ to 1 × 10^−7^ (mutations per bp). Some studies (e.g., R and V) exhibit a broad spread of MFs with large variability, whereas others (e.g., F to M) have tighter clustering. Study V is a repeat of study U; to ensure independence, study U was excluded from all subsequent analyses.

**FIGURE 2:**
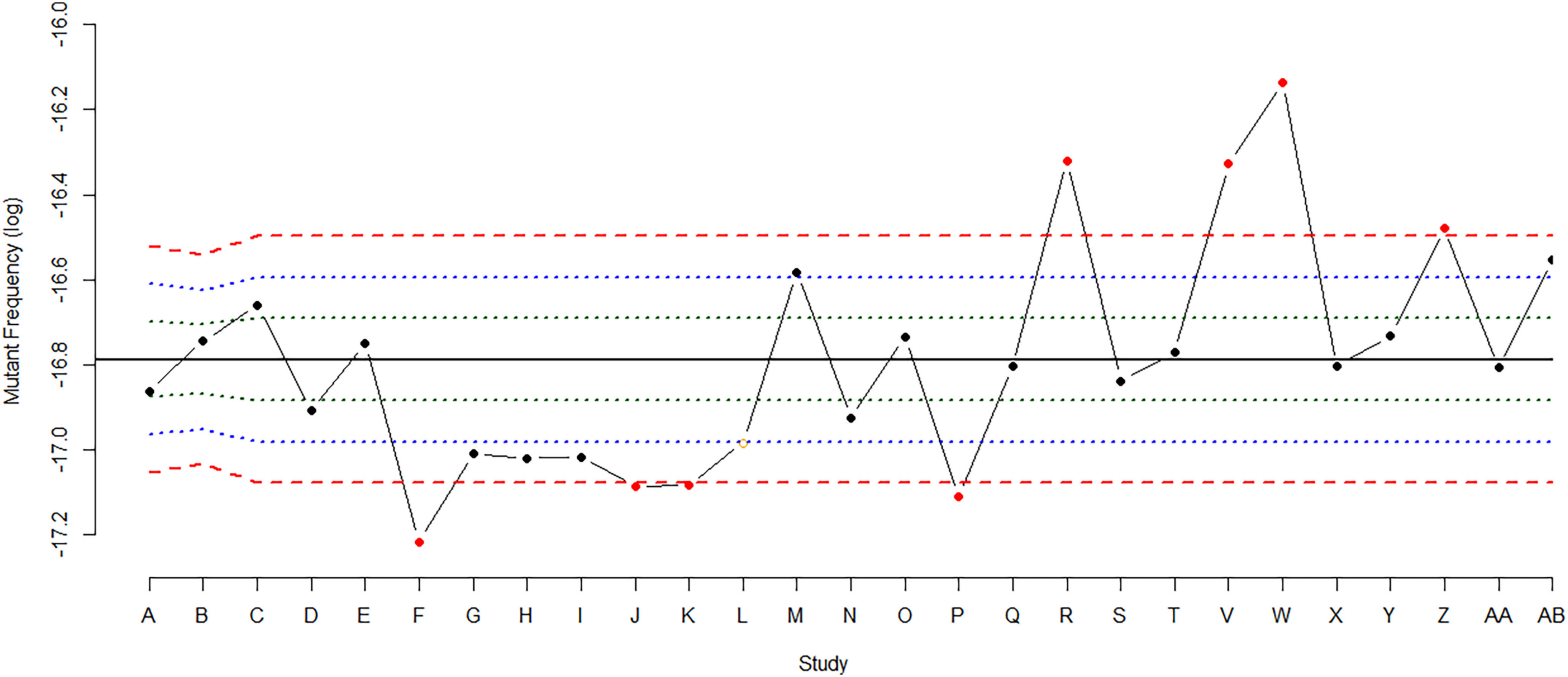
Control chart of vehicle control data. The *X̄* chart plots the mean of the natural logarithm of mutation frequency for each study (A–AB) on the *x*‐axis. The solid black line indicates the overall mean. The red dashed lines represent the upper and lower 3‐standard deviation (3*σ*) control limits; the blue and green dotted lines represent the 2‐ and 1‐standard deviation control limits, respectively, from the mean. Red points denote studies outside the 3‐sigma limits (out‐of‐control); the one orange open circle indicate a study violating the runs rules. Note that the control limits for Study A and B are tighter due to the absence of a preceding value for calculating the moving range, which is standard in Shewhart chart methodology.

We produced control charts to further display and assess our data (Figure 2). Applying the Shewhart rules (Nelson 1984; Western Electric Company 1956), studies with a red dot glyph (F, J, K, P, R, V, W, Z) exceeded the 3‐sigma control limits. Notably, some studies (J, K, P, and Z) are only slightly beyond the 3‐sigma threshold, while F, R, V, and W exceeded it by a larger margin. One study is labeled with an orange dot (Study L), which signifies a violation of a runs rule (i.e., seven or more consecutive points on one side of the mean; two out of three consecutive points outside the 2‐sigma limits; or four out of five consecutive points outside the 1‐sigma limits). The relatively large number of studies exceeding the 3‐sigma control limits, along with the occurrence of extended sequences below the mean (as in Study L), suggests that the overall variance of the data is underestimated by the current model and strongly indicates higher study‐to‐study variation.

To estimate type 1 error rates, we performed six analyses representing different levels of animal‐to‐animal variation. These analyses were structured to successively exclude studies as follows: (1) using all available data; (2) removing study W, which was the furthest from the overall mean; (3) additionally removing study R (i.e., both W and R excluded); (4) further removing study V (i.e., W, R, and V excluded); (5) considering only the first 12 studies (A–L); and (6) considering studies M–AB, excluding W, R, and V. For each analysis, it is assumed that mice are independent and are drawn from the same underlying distribution. Under this assumption, the estimated sample‐level standard deviations (on the log MF scale) from the variance component analysis were 0.2857, 0.2565, 0.2267, 0.2027, 0.1684, and 0.1940, respectively. As expected, stepwise removal of studies with MF values outside the quality control limits led to a progressive reduction in overall variance and the first 12 studies had lower sample variation compared to the last 12 studies. Using these values in the simulation analysis allows for direct comparison to the empirical evaluation performed here. For the simulations, we used a vehicle control MF of 5.02 × 10^−8^ and 8.8 × 10^8^ informative duplex bases, matching the median values from the control dataset. The simulated data were analyzed using the same statistical approach as the experimental data to estimate the observed type I error rate. Using these values in the simulation analysis allows for direct comparison to the empirical evaluation performed here.

We note that, in our analysis, exclusion of different outlier studies (including study F) did not materially affect the observed type 1 error rates, which remained consistent across all data subsets. We recognize that rigorous monitoring and curation of historical control data are critical for assay quality assurance and regulatory use, but a comprehensive evaluation of historical control management is beyond the scope of the present study.

**FIGURE 3:**
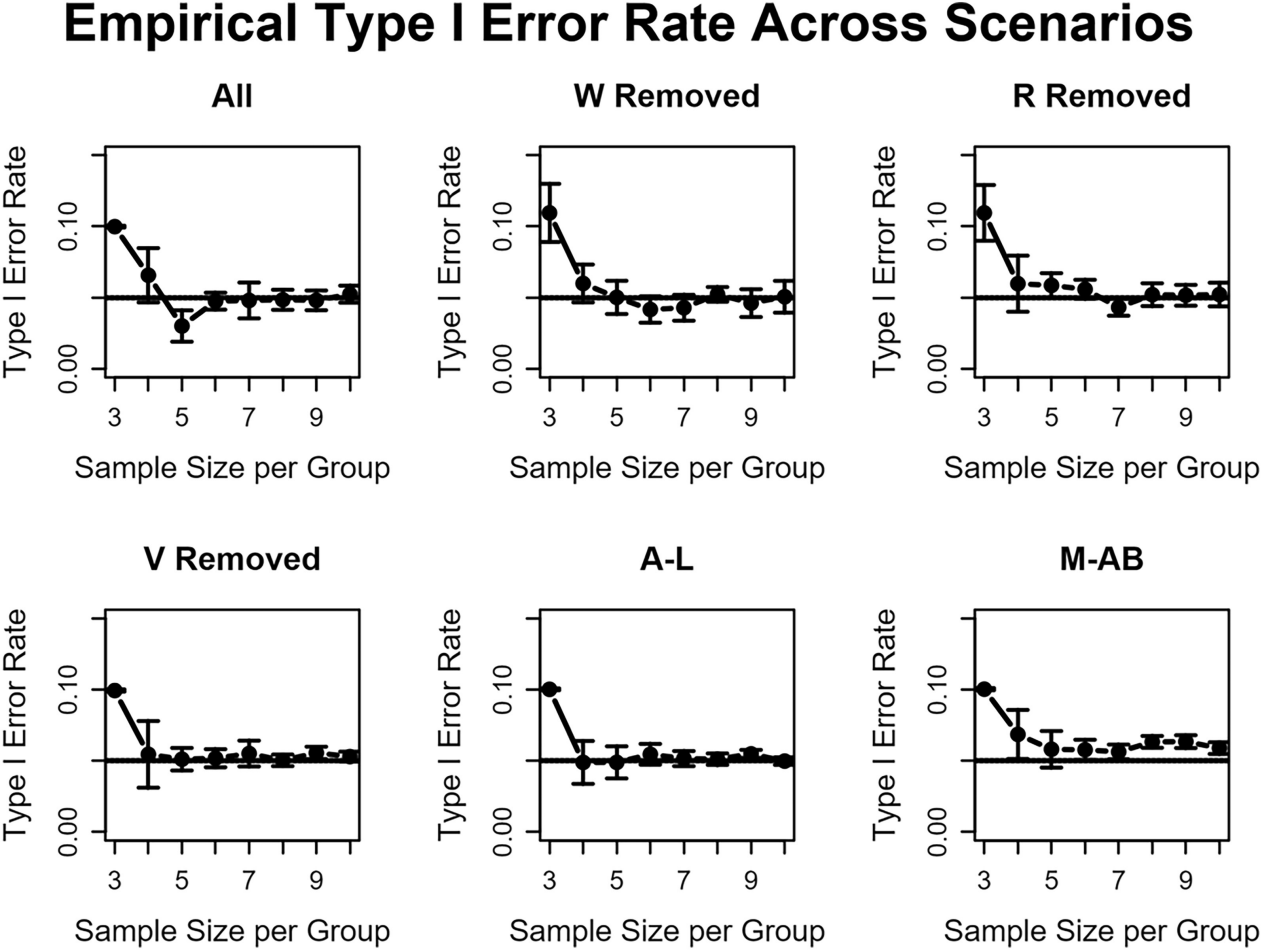
Type I error rates as a function of sample size per group for each empirical analysis scenario. Each panel represents a different data inclusion/exclusion scenario: (1) all studies, (2) W removed, (3) R removed, (4) V removed, (5) studies A–L only, and (6) studies M–AB excluding W, R, and V. Error bars denote standard errors. The solid horizontal line marks the nominal 0.05 type I error threshold.

**FIGURE 4:**
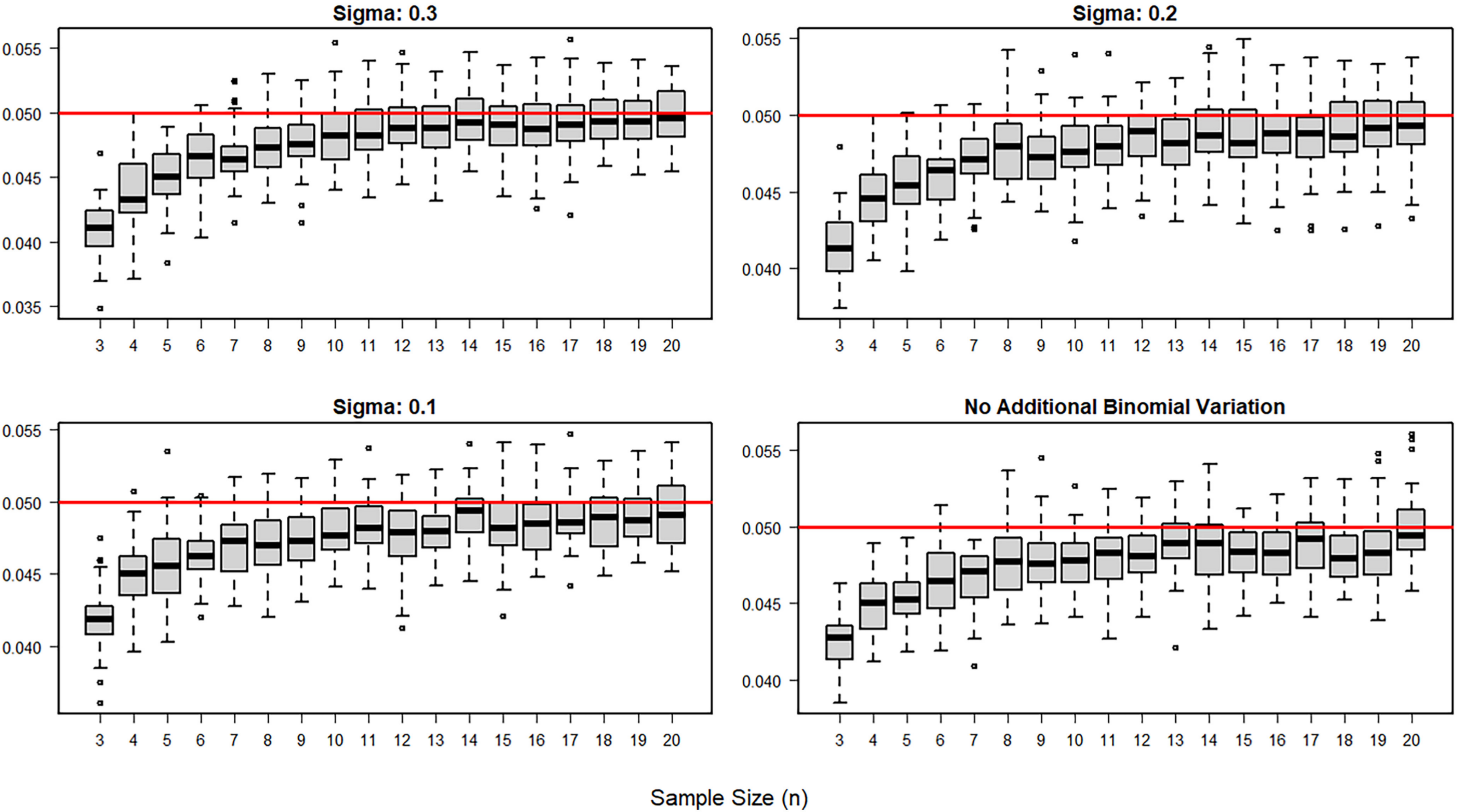
Impact of overdispersion on the distribution of type I error rates estimated across increasing sample sizes (*n* = 3–20). Each panel corresponds to a different level of overdispersion (*σ* = 0.3, 0.2, 0.1, or no additional binomial variation). For each scenario, boxplots show the distribution of 20 simulated type I error rate estimates for each sample size. The central line of each box indicates the median; boxes denote the interquartile range; whiskers extend to 1.5× the IQR; and points represent outliers. The red horizontal line marks the nominal significance level of 0.05 used for all tests. Type I error rates are slightly lower and more variable at small sample sizes, increase with n, and stabilize for moderate to large n across all overdispersion scenarios, with overall control of the type I error rate maintained near the nominal level.

For sample sizes of four or more per group, type I error rates were generally well‐controlled. Across all four empirical scenarios with different levels of animal level variability, the observed type I error rate did not exceed the nominal 0.05 threshold for group sizes of four or greater. In contrast, when only three samples per group were used, the empirical type I error rate rose to approximately 0.10 across all scenarios. Figure 3 summarizes the empirical error rates, while Figure 4 shows the boxplots of the simulation‐based results. In nearly all cases, the average empirical error rate across the 10 replicate simulations remained below 0.065, and the type 1 error rate remained below 0.06 for the simulation analysis, with a few exceptions under higher overdispersion. These findings suggest that while a sample size of four is generally sufficient, under conditions of increased overdispersion, sample sizes of five or six may be required to maintain type I error rates near the nominal 0.05 threshold.

Further exploration of the sample data distributions revealed that the discrepancy between empirical and simulated type I error rates at small sample sizes is primarily attributable to the distributional structure of the empirical vehicle control data. In particular, the empirical data exhibit evidence of multimodality reflecting the underlying study‐to‐study heterogeneity, whereas the simulated data are unimodal by design (Figure S4). This multimodality means that, when randomly assigning small groups (e.g., *n* = 3) for mock control and treatment, it is more likely that each group will be drawn from distinct underlying subpopulations in the empirical data, inflating the type I error rates. By contrast, the simulation models assume a single underlying distribution, which may smooth over real biological or technical heterogeneity.

A previously published power analysis demonstrated that a sample size of four is sufficient to achieve adequate statistical power for most observed sample‐level variances (Esina et al. 2024). Increasing the sample size to five extends this capability to scenarios with variability up to a standard deviation of 0.3. In that work, analyses using real data confirmed these calculations. To detect a minimum 1.5‐fold change in MF, sample sizes of four to five were adequate, even under high sample‐level variation with the enzymatic fragmentation protocol in MutaMouse liver and bone marrow. Although fold‐change thresholds are not formal design criteria in traditional OECD mutagenicity assays, a two‐fold increase is commonly used as a benchmark for interpreting biological relevance (OECD 2017). In this context, the ability of DS to detect smaller effects (e.g., 1.5‐fold) illustrates its potential for increased sensitivity.

Taken together, the findings from our study of type 1 error rate control using data from Big Blue mice, combined with the conclusions of Esina et al. (2024) regarding statistical power and sample size in MutaMouse DS studies, suggest that a minimum sample size of four per group is generally sufficient to balance type 1 error control and statistical power. Increasing the sample size to five may extend this capability to scenarios with higher variability. While fold‐change thresholds required in traditional OECD mutagenicity assays, both studies indicate that DS can sensitively detect effects as small as a 1.5‐fold increase in MF. These complementary analyses support the reliability of recommended sample sizes for DS‐based mutagenicity assessment.

There are several limitations to this analysis. First, our evaluation focused exclusively on Big Blue mouse liver tissue. MFs and sample‐level variation can differ in other tissues or species (Smith‐Roe et al. 2023; Schuster et al. 2024), which may limit generalizability. An additional limitation is that Big Blue is an inbred mouse line. Outbred stocks, such as Sprague Dawley rats, may exhibit greater genetic diversity, potentially leading to increased biological variability in MF measurements. However, we note that the power analyses produced by Esina et al. (2024) used MutaMouse, an outbred model. Nonetheless, greater genetic variation in outbred lines could influence the number of samples needed to reliably control type 1 error rates, suggesting the need for additional studies to estimate type 1 error rates for ECS applied to outbred models. Importantly, the simulation framework itself is flexible and can be adapted to other experimental designs and assumptions. Second, the findings are specific to DS as implemented in this study; other ECS platforms may exhibit different performance characteristics. Third, the variance component analysis assumes normality and independence of random effects; violations of these assumptions could affect the reliability of variance estimates. Fourth, although Shewhart control charts are widely used for identifying outliers, alternative methods may yield different results. Fifth, while our simulations were informed by empirical vehicle control data, they necessarily relied on point estimates for MF and sample‐level variation, which may not fully capture the broader range of variability observed across real‐world datasets.

While our study focused on type 1 error rates based on MF, it is worth noting that ECS simultaneously provides detailed information on the types and patterns of mutations (the mutation spectrum). Incorporating mutation spectrum analysis may offer additional power to distinguish true mutagenic effects from random variation or technical artifacts, and thus could be used to further reduce type 1 error rates. We recommend that future studies explore how mutation spectrum data can be used alongside MF to increase the specificity and reliability of ECS‐based mutagenicity assays.

While historical control data were instrumental for this analysis and are likely informative for other tissues and applications, generating such datasets may not always be feasible or necessary. Ongoing experience with DS across diverse systems (e.g., different tissues or animal models) will help clarify when extensive historical data are warranted and when more limited control data are sufficient to ensure assay reliability. Detection of out‐of‐control experiments through vehicle control monitoring provides an important tool for assay quality assurance. Occasional outliers are expected in any biological assay due to intrinsic variability among animals, experimental handling, or unrecognized biological factors. The use of control charts and vehicle control monitoring provides a transparent and objective framework for identifying such outliers, without implying that they necessarily reflect a flaw in the assay itself or its suitability for regulatory use. For regulatory applications, predefined rules for data acceptance and criteria for repeating experiments should be established by the expert community, consistent with standard practice in other OECD test guidelines.

The considerable inter‐study variation observed in the present dataset highlights that historical control data (HCD), where available, can provide valuable context for interpreting DS results similar to “criterion c” considerations in established OECD in vivo genotoxicity assays (OECD TG 488 2025a, OECD TG 470 2025b; Dertinger et al. 2023). As with conventional assays, HCD may aid in identifying atypical control values, contextualizing borderline responses, and interpreting small increases in MF relative to expected biological and technical variability. However, the absence of an extensive HCD should not be regarded as a prerequisite for the validity or regulatory utility of DS studies. A key strength of DS is its applicability across a wide range of tissues, species, and experimental designs, including novel targets where historical datasets may not yet exist. In such situations, interpretation must rely on robust concurrent controls, transparent quality metrics, and suitable statistical frameworks, with HCD serving as a complementary rather than determinative line of evidence. As DS technologies mature, systematic compilation and sharing of control data will broaden the availability of qualified HCD, thereby supporting harmonization and confidence in regulatory mutagenicity testing, yet without constraining scientific exploration or innovation.

## 4 Conclusions

Study designs should align with the biological characteristics of the test system and the specific goals of the mutagenicity assessment. Our results show that, in mouse liver DS studies, the type I error rate is controlled at the desired level when there are at least 4 samples per group. OECD TG 488 states that sample sizes should be predetermined to provide sufficient statistical power to detect at least a doubling (2‐fold increase) in mutant frequency (MF), with a recommended minimum group size of five animals per group (OECD 2017). In our analyses, sample sizes of three to four per group were generally sufficient to detect a 2‐fold change, whereas sample sizes of four to five were required to reliably detect a minimum 1.5‐fold change in MF, consistent with previous findings (Dodge et al. 2023; Esina et al. 2024; Zhang et al. 2025).

However, it is important to note that while our simulation study suggested type I error rates were at or below the nominal level for very small sample sizes (*n* = 3–4), empirical results revealed type I error rates substantially above nominal, with values near 0.1 when *n* = 3. This discrepancy likely reflects departures from the simulation assumptions in real‐world data such as greater heterogeneity, unaccounted overdispersion, or other data complexities. Thus, we caution that simulation‐based error rates may underestimate actual risks in the smallest groups and recommend empirical validation whenever possible.

The vehicle control data used in this study were an exceptional resource. Vehicle controls remain essential for DS analyses to improve accuracy in estimating background MFs and sample level variations in different tissues. Vehicle control data can also be used for quality assurance of the test system; for identifying abnormal controls and experiments; to help in distinguishing true responses from chance findings; to judge biological relevance; and to informally address the statistical multiple comparison problem (Kluxen et al. 2021). We strongly recommend the collection and publication of similar data across more model systems and tissues, and ensuring public availability to the full data sets for more in‐depth analyses such as the one herein. As more data become available, similar analyses could extend to understand ability to detect changes in mutation spectra, further informing suitable approaches for integrating DS and similar ECS technologies into regulatory testing.

## Author Contributions

**Andrew Williams:** methodology, formal analysis, writing – original draft, writing – review and editing, conceptualization. **Shaofei Zhang:** methodology, data curation, writing – review and editing. **Dingzhou Li:** feedback, writing – review and editing. **Wen Sun:** data curation, writing – review and editing. **Maik J. Schuler:** data curation, writing – review and editing. **Francesco Marchetti:** conceptualization, writing – review and editing. **Carole L. Yauk:** conceptualization, writing – review and editing. All authors read and approved the final version of the manuscript.

## Funding

This work was supported by Canada Research Chairs (CRC‐2020‐00060) and Burroughs Wellcome Fund (1021737).

## Conflicts of Interest

The authors declare no conflicts of interest.

## Supporting information


**Table S1:** Supporting Information.

**Table S2:** Supporting Information.

## Data Availability

The data that support the findings of this study are available from the corresponding author upon reasonable request.
